# Nanoparticle drug delivery systems: an excellent carrier for tumor peptide vaccines

**DOI:** 10.1080/10717544.2018.1477857

**Published:** 2018-06-05

**Authors:** Jiemin Wang, Xiongbin Hu, Daxiong Xiang

**Affiliations:** a Department of Pharmacy, Second Xiangya Hospital Central South University, Changsha, Hunan Province, China;; b Institute of Clinical Pharmacy Central South University, Changsha, Hunan Province, China;; c Hunan Provincial Engineering Research Center of Translational Medicine and Innovative Drug, Changsha, Hunan Province, China

**Keywords:** Drug delivery system, immunotherapy, nano, peptide vaccine, tumor

## Abstract

In the past 40 years, the nanoparticle drug delivery system for tumor peptide vaccines has been widely studied which also reached a splendid result. Nanomaterial can enhance the targeting of vaccines, help vaccines enter the cells and trigger immune response by themselves. They also help in increasing cellular uptake, improving permeability and efficacy. Currently, several categories of nanopreparation, such as liposome, polymeric micelle, polymeric nanoparticle, gold nanoparticle and so on, are proved that they are appropriate for peptide vaccines. This review we discussed the possible mechanisms of nanomaterial’s action on the regulation of immunological functions and several major applications of this advanced drug delivery system for tumor peptide vaccine.

## Introduction

Nowadays, the morbidity and mortality of therioma was hard to be controlled. Although the traditional therapeutic method such as surgical operation, radiotherapy and chemotherapy has been improved. It seems still difficult to further enhance the survival rate of patients with tumor. Meanwhile, the combination of the anti-tumor immunotherapy and the traditional therapy is becoming the new trend of prospective strategy to treat tumor (Bhargava et al., 2013; Melero et al., 2015; Vonderheide et al., 2017).

Anti-tumor immunotherapy aims to enhance the immunity function by artificial method, sequentially inhibit the growth of tumor cells, and eventually remove the tumor cells (Singh & Peppas, 2014). As one important constituent of the anti-tumor immunotherapy, the tumor vaccine has been becoming more and more promising in researches. The tumor vaccine is the method which utilize tumor antigen, immunocyte or other immune molecules to activate the immune system and induce immune response (Yue et al., 2015). According to the origin of tumor vaccine, it can be divided into several categories: tumor cell vaccine, dendritic cell (DCs) vaccine, peptide vaccine, gene vaccine and so on (Li C et al., 2015).

Researches have shown that the immune system can discriminate between cancerous and normal cells, because the tumor cells have specific tumor antigens. The tumor peptide vaccine can directly activate or strengthen anti-tumor immunity in the body to kill and eliminate tumor cells by the expression of the tumor antigen of immunogencity with the assistance of cytokine, chemotactic factor and other adjuvant. It can also induce high immune response, and does not cause autoimmunity and immune suppression (Sen & Mandal, 2013). Additionally, the low carcinogenic risk is another advantage of it (Prasad et al., 2011). However, the deficiencies like weak immunogenicity and short half-life period exist as well (Mocan et al., 2015). This kind of tumor vaccine is easy to produce immune tolerance, and the major histocompatibility complex (MHC) restriction and relatively low bioavailability of the drug are also need to be overcome (Melero et al., 2015; Wang et al., 2016).

**Figure 1.**
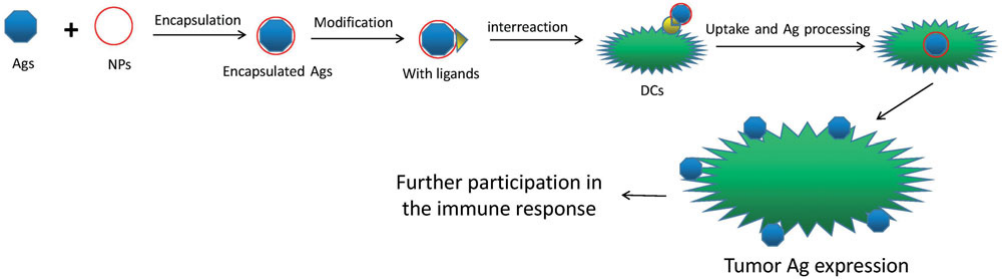
Sketch of mechanism of immunotherapy.

Therefore, what need to be emphasized specially is that the tumor peptide vaccine would achieve a better effect via nano drug delivery system (Figure 1). In the past 30 years, the nano drug system for tumor peptide vaccine has been widely studied which also reached a splendid result. First of all, it is important that the NPs allow target specificity to miminize the side effect of treatments (Delie et al., 2012; Valente et al., 2017). Secondly, some of nanoparticles are easy to be modified to express plenty of good properties like stability for a better release and absorption of drug molecule (Harms & Mueller-Goymann, 2011). Thirdly, it could protect the antigen from being degraded and removed quickly that could prolong the active time of antigen, and sequentially enhance the efficiency of uptake and delivery of antigen (Chiang et al., 2013). Fourthly, it also could play an adjuvant role on improving the immune effect and therapeutic consequence of tumor vaccine in many ways (Friede & Aguado, 2005). Last but not least, it could achieve the constransmitter of tumor vaccine and immunopotentiator, make the both act on the same antigen presenting cells (APCs) (Li et al., 2011), and achieve a synergistic action (Vangasseri et al., 2006; Klippstein & Pozo, 2010; Pushpalatha et al., 2017).

Currently, that making nanoparticles as the carrier of tumor vaccine, which target the immune system, provide the opportunities for the better immunization. This review we discussed the possible mechanisms of nanomaterial’s action on the regulation of immunological functions and several major applications of this advanced drug delivery system for tumor peptide vaccine.

## The possible mechanisms of nanomaterial for enhancing the effect of vaccines

It is summarized that delivery systems in peptide vaccines should actively or passively target APCs such as DCs, protect the peptides from degradation if taken orally and induce APC maturation by interacting with elements of the innate immune system such as Toll-like receptors (TLRs) (Bolhassani et al., 2011). Due to the special characteristics, nanomaterial or nanotechnology hold the potential to meet the above requirements. What’s more, nanomaterial can act as adjuvant or carrier of a therapeutic or prophylactic vaccine. And can also bear several antigenic substances simultaneously to facilitate multivalent vaccines. The biological activity of vaccines may be promoted with the help of nanomaterial (Liu & Chen, 2016).

**Figure 2.**
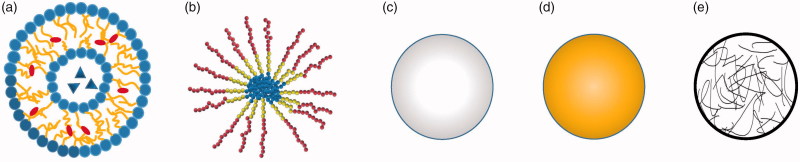
Representative structures of various NPs for drug delivery. (a) Liposomes, (b) polymeric micelles, (c) polymeric nanoparticle, (d) gold nanoparticle and (e) nanogel.

**Table 1.** The advantages and disadvantages of each kind of nanoparticle for peptides delivery.

Categories	Advantages	Disadvantages	References
Liposome	✔ Long circulation time. ✔ High drug loading. ✔ Can be equipped with targeting ligands.	× Partly lose the ability to tailor liposome characteristics for optimal drug retention and release profiles. × Decrease targeting capacity owing to nonspecific serum protein binding, and hinder tumor penetration.	Noble et al., 2014; van der Meel et al., 2014
Polymeric micelles	✔ Lessen the P-glycoprotein (P-gp) efflux effect. ✔ Improved stability. ✔ High drug loading.	× Tend to dissociate and release payloads upon extensive dilution and interaction with proteins and cells in the blood pool, which often lead to premature drug release following i.v. injection.	Gong et al., 2012; Zhong et al., 2014
Polymeric nanoparticle	✔ Release a drug at a rate controlled. ✔ Allow encapsulation of bioactive molecules and protect them against enzymatic and hydrolytic degradation.	× Too sophisticated to put on production, while the simple cannot meet the curative requirement.	Hsieh et al., 2016; Ulbrich et al., 2016; El-Say & El-Sawy, 2017
Gold nanoparticle	✔ High degree of stability and biocompatibility. ✔ Be multi-functional after modification. ✔ Can be combined with radiotherapy or photodynamic therapy.	× Multi-functionalized GNPs become increasingly more complicated and balance must be achieved between suitable *in vivo* stability, tumor localization, limited cytotoxicity and efficacy.	Kogan et al., 2007; Nicol et al., 2015
Nanoemulsion	✔ Micro-domain of different polarity within the same single phase solution can facilitate solubility of hydrophilic or lipophilic peptides. ✔ Usually be designed as oral dosage forms for convenience.	× More mechanistic studies are needed to shed new insights on the understanding of the interplay between nanoemulsion, peptides and physiological conditions in the intestine. × Bio-relevance must receive more attention	Niu et al., 2016
Nanogel	✔ The formation by covalent crosslinking reactions induces the stability in a complex and unkind environment, preventing the leakage of the encapsulated drug. ✔ High drug loading.	× Traditional covalent crosslinking usually involves crosslinking agents, which may cause unwanted toxic effects and damage the entrapped peptides.	Neamtu et al., 2017

### Nanomaterial enhances the targeting of vaccines

The most important effect of nanomaterial or nanotechnology is targeting, which has been widely used, studied, illustrated. Targeted therapy of nanomedicine get used of nanoparticles as the carrier to distribution of drug, and make the drug molecular only effect on the diseased tissue or target cell, which avoid the effect on normal cells (Ahmed & Aljaeid, 2016). The ideal design is to insert antibodies on the surface of nanoparticle that can bind to the membrane proteins of specific cancer cells so that they can act as ‘missiles’ (Dinda & Pattnaik, 2013; Huo et al., 2017). Specifically, the nanoparticle has some good properties like tropism of lymphatic system, passive targeting (easy to be engulfed by macrophages as foreign bodies), active targeting (by modification) and physical targeting (by encapsulating magnetic material).

### Nanomaterial itself triggers immune responses

Synthetic nanomaterial have many unique chemical and physical properties, mainly due to their high specific surface area and quantum confinement effect. Some researchers find that nanomaterial itself triggers immune responses. Sun & Xia (2016) listed several mechanisms of immune responses which nanomaterial can initiate dynamic which relate to (i) depot effect, (ii) NLRP3 inflammasome activation, (iii) perturbation of DCs membrane, (iv) autophagic regulation, (v) lymph node targeting, (vi) toll-like receptor signaling, (vii) B cell activation, (viii) T cell differentiation, (ix) antigen presentation, (x) host DNA release, and (xi) soluble mediators. Therefore, understanding the molecular mechanisms of immune activation is critical in rational design of engineered nanomaterial for optimal and long lasting immuno-potentiating effects. Nanomaterials do play an important role in directing immunity towards either a bacterial or viral defense pathway.

However, we need to pay attention to the drug adverse effect of nano drug by extreme immune response. Leading allergy, mast cell degranulation or other adverse effect, some NPs, like titanium dioxide nanofiber (Gato et al., 2017), silica NPs (Marquardt et al., 2017) and silver NPs (Alsaleh et al., 2016), are widely researched for a better usage.

### Nanomaterial increases cellular uptake of vaccines

As a novel drug delivery system, due to their small size and high surface area, nanomaterial can interact with biomolecules both at the surface and inside cells easily. On the other hand, after the artificial modification, the structure of whole drug has changed, which is beneficial for the uptake and the induction of immune response (Hu et al., 2015; Wang et al., 2016). Therefore, the NPs for cells, such as DCs, were used for the delivery of encapsulated antigen, which resulted in a much more efficient uptake of soluble antigen (Bharali et al., 2008; Klippstein & Pozo, 2010).

To prove this principle, a tissue-like pulmonary surfactant monolayer (PSM), can set a good example. Xu et al. (2017) used PSM which act as the first barrier to demonstrate the transport mechanism of NPs. They study how inhaled NPs interact with the PSM. The results showed that the binding energy which drives transport can be strengthened by increasing the lipid coating density and the lipid tail length. So the binding energy is factor which increase the cellular uptake. For animal models, the principle also exist. To study *Leishmania panamensis*, researcher set a model of mice, which treated with free CpG, empty NPs and NP-CpG. Surprisingly, the NP-CpG led an unexpected preferential cellular uptake at the site of infection. So combined with NPs, the vaccines could be more effective (Siefert et al., 2016).

What need to concentrate on is several special method to facilitate the cellular uptake. The biological linkage such as avidin–biotin interactions are coupled with nanocarriers for a better cellular uptake (Jain & Cheng, 2017). For instance, paramethoxyam–phetamine hydrogel capsule functionalized with biotin which finally turn into a stable nanocomplex with avidin-coupled antibodies, and express its better cellular uptake in cancer cells (Shimoni et al., 2012). In addition, the electrically activated plasmonic gold nanoparticles (GNPs) have the unique properties. Their electrical excitation can drive vibrational and dipole-like oscillations, which are able to disrupt nearby cell membranes. In the research, mice immunized with this approach showed up to 100-fold higher uptake compared to control treatments which is without NPs (Draz et al., 2017).

### Nanomaterial increases permeability and efficacy of drugs passing through biological barriers

In this review, we discussed three kinds of barriers which are mucosal barrier, skin barrier and blood–brain barrier (BBB). The nanomaterial can improve the drug’s permeability to overcome them.

For mucosal barrier, the nanoparticles can prolong the retention time of gastrointestinal tract, synchronously avoid the drug being destroyed by the harsh environment of enzyme and sequentially improve the absorption of the drug by electrostatic interactions, hydrophobic interaction, van Edward and polymer chain interaction (Lai et al., 2009; Bernocchi et al., 2016). Specifically, Popov et al. (2016) designed nanoparticles coated with poly vinyl alcohol (PVAs) that are <95% hydrolyzed. This NPs were able to penetrate mucus with velocities significantly exceeding those for the mucoadhesive controls. Meanwhile, Ji et al. (2017) designed nanocomplexes of recombinant adenovirus (rAd) particles coated with (i) the polyethylene glycol (PEG) to provide a hydrophilic surface that would prevent entrapment in the hydrophobic mucus, and (ii) the cell-penetrating peptide TAT to improve transduction efficiency. The optimized nano-complexes could penetrate the mucus barrier to a much greater extent than their previous work without nanoparticle.

For skin barrier, transdermal drug delivery has made a significant contribution in the management of skin diseases with enhanced therapeutic activities over the past two decades. What deserve to be mentioned is that nanomaterial plays an important role in the transdermal delivery, due to its ability to help the drug permeate the stratum corneum which is regarded widely as the main barrier (Kotla et al., 2017). As a part of nano drug, nano-emulsions also can increased permeability of drug to overcome skin barrier. Burger et al. (2017) summarized that nanoemulsions have a number of advantages over conventional emulsions, which include easy preparation using various low and high energy methods, the ability to penetrate the skin, high solubilization capacity, high stability to droplet aggregation and optical transparency. Thus allowing the transdermal delivery of drugs. Additionally, molecular level understanding of permeation of NPs is studied by Gupta & Rai (2017). They carried out coarse grained molecular dynamics simulations to explore the permeation of dodecanethiol coated neutral hydrophobic GNPs of different sizes (2–5 nm) and surface charges (cationic and anionic) through the model skin lipid membrane. He concluded that there is a maximum permeability with specific particle size and surface charge of GNPs, which contribute to the design of transdermal drug delivery application. Maybe, this principle can expanded to all NPs.

For BBB, it makes drug infusion through central nervous system challenging with the truth that the patients with brain tumor or brain metastases are not uncommon (Dinda & Pattnaik, 2013). This review listed three possible mechanism of NPs for entering BBB. (i) NPs are used as carrier to adsorb drugs on cerebral capillary wall, prolong the residence of drugs in the absorption sites, and enhance the gradient concentration of drugs inside and outside the blood vessels, which is beneficial to the drug entering the brain. (ii) NPs produce surface activity, increases the solubility of lipid in vascular endothelial cell membrane, and lead to the increase of membrane permeability to drugs. (iii) Drug-loaded NP is engulfed by cerebral vascular endothelial cells, releasing drugs and transferring them into the brain (Kreuter, 2005, 2012). As an example, a reported engineered liposome with carrying four drug combinations (temozolomide, procarbazine, doxorubicin and carmustine) for glioma treatment increases 10-fold efficacy when compared to other methods (without liposome) in cell model (Zhu et al., 2017). Besides, two reports about curcumin for entering BBB can also manifest how strong the NPs are. One is encapsulated in PLGA (Barbara et al., 2017) and modified with g7 ligand. The other is encapsulated in PLGA-DSPE-PEG (Orunoglu et al., 2017). This two curcumin-loaded NPs showed a good permeability to get across the BBB. Applications of nano drug delivery system for tumor vaccine.

## Examples of applications in nano peptide tumor vaccines

The advantages and disadvantages of each kind of NPs for peptides delivery are listed (Table 1) and representative structures of major NPs are summarized (Figure 2).

### Liposome

Liposome have a good effect on cancer. Because it has dual characteristics of hydrophilic and hydrophobic, which can effectively bind water-soluble and fat-soluble substances (Jia et al., 2013). As a tumor vaccine carrier, liposome can achieve vaccine targeting delivery and cross presentation, it can also encapsulate tumor antigens, and significantly enhance the tumor specific immune response to vaccines and immune effect of the body.

The research team of Jaafari have focused on this area for many years. Early in 2012, they demonstrated p5 peptides with the lipid-protamine-DNA NPs produced a considerably higher IFN-γ and cytotoxic T lymphocyte (CTL) responses compared to free peptides (Jalali et al., 2012). Two years later, they change the p5’s “partner” and also have a great effect. On the one hand, they made p5 peptide coupled with maleimide-PEG2000-DSPE and combined by liposomes which contains monophosphoryl lipid A (MPL, as adjuvant) (Shariat et al., 2014). On the other hand, they used cationic liposomes which are comprised of 1,2-dioleoyl-3-trimethyl ammonium propane (DOTAP)-cholesterol to enhance the cytosolic delivery of p5 HER-2/neu generated peptide which acted synergistically as the antigen (Mansourian et al., 2014). Both of the improved liposomes above stimulate higher provocation of Th1 (IFN-γ) responses than peptides in buffer and nanoparticles without adjuvant. Then concluded that the liposomes could impressively increase the CTL response and substantially restrain tumor growth. On the basis of Mansourian, making use of chaotropic transfer technique, new researches from Yazdani et al. (2017) made liposomes with high transition temperature (T_m_) to sustain the stability, in the function of a vaccine for P5 HER2/neu generated peptide. After evaluation, especially in experiments *in vivo* the results suggested that immunization with Lip-p5 nanoliposomes has enhanced the antigen-specific IFN-γ response of CD8^+^ T cells and induced CTL response in comparison with free peptides and empty liposomes, which resulted in a smaller tumor and longer survival time. In Jaafari’s team, the cytotoxic T-lymphocyte-associated protein 4, AE36 HER2/neu-derived peptide and PNC peptide are also studied as the antigen (Barati et al., 2017; Darban et al., 2017; Nikpoor et al., 2017). What’s more, the adjuvant which can enhance and prolong the immune responses with liposome like cyclic dinucleotides guanosine monophosphate (c-di-GMP) and MPL should be focused on too (Miyabe et al., 2014; Shariat et al., 2014).

### Polymeric micelle

As a drug delivery system, polymeric micelle is a thermodynamically stable colloidal solution formed by self-assembly of amphiphilic block copolymer. And it has many advantages, such as enhancing drug stability, delaying release, enhancing efficacy, targeting and reducing toxicity.

Especially for metastatic melanoma, polymeric micelle may have the potential to treat it. Zeng et al. (2017) tailor the physicochemical properties of polymeric hybrid micelles (HMs), which are self-assembled from two amphiphilic diblock copolymers, poly(ethylene glycol)phosphorethanolamine (PEG-PE) and polyethylenimine-stearic acid conjugate (PSA), to address lymph nodes via hydrophobic and electrostatic interactions. In detail, they successfully encapsulated melanoma antigen peptide Trp2 and TLR-9 agonist CpG ODN into HMs to low efficient delivery of antigen/adjuvant to secondary lymphoid organs. The results were that the polymeric HMs expressed higher targeting and better curative effects than free Trp2 and mixture of Trp2 and CpG. This study also contribute to more rational design of nanoparticles-based cancer vaccine platforms.

Last but not least, not like common chemotherapy, the polymeric micellce may enhance therapy for advanced tumor by remodeling the tumor microenvironment (TME). Huo et al. (2017) encapsulated a multi-target receptor tyrosine kinase inhibitor into a targeted polymeric micelle nano-delivery system, working in a synergistic manner (along with sunitinib) with vaccine therapy in an advanced mouse melanoma model. Finally, cytotoxic T-cell infiltration increased and the number and percentage of MDSCs and T regulator cells (Trgs) in the TME decreased, and treated with peptide vaccine and sunitinib, the tumor (*in vivo* and *in vitro*) slowed down the progression in comparison with the group used single drug, which indicated that targeted delivery of a tyrosine kinase inhibitor to tumors can be used in a novel synergistic way to enhance the therapeutic efficacy of existing immune-based therapies for advanced melanoma.

### Polymeric nanoparticles

Polymer-based NPs are submicron-sized polymeric colloidal particles in which a therapeutic agent of interest can be embedded or encapsulated within their polymeric matrix or adsorbed or conjugated onto the surface (Labhasetwar et al., 1998; Mahapatro & Singh, 2011). It can effectively improve the immune responses. Polymer properties such as biocompatibility, low toxicity and biodegradability have highlighted polymeric NPs as an interesting delivery strategy (Conniot et al., 2014).

Similar to polymeric micelles, the first great advantage of using polymeric NPs is to allow encapsulation of bioactive molecules and protect them against enzymatic and hydrolytic degradation. For instance, it has been found that insulin-loaded NPs have preserved insulin activity and produced blood glucose reduction in diabetic rats for up to 14 days following the oral administration (Damge et al., 2007; Mahapatro & Singh, 2011).

Meanwhile, polymeric NPs do enhance the immunity. Luo et al. (2017) reported a minimalist nanovaccine, which comprise a simple physical mixture of an antigen and synthetic polymeric NPs, PC7A NP, which generates a much stronger cytotoxic T-cell response with low systemic cytokine expression than using antigen alone. In his meticulous research, the PC7A used ovalbumin (OVA) as a model antigen whose loading efficiency was measured to be >75% for different candidate polymer NPs. After his across-the-aboard experiments *in vitro* and *in vivo*, the conclusion is that the polymeric NPs led to potent tumor growth inhibition in melanoma, colon cancer and human papilloma virusE6/E7 tumor models. Besides, the combination of the PC7A nanovaccine and an anti-PD-1 antibody also showed great synergy.

Lastly, this approach that building a polymeric nanoparticle model has a tendency to merge with other methods to achieve a better efficacy in the recent time. Except synergizing with anti-PD-1 antibody above, the example is about the design of biohybrid drug delivery systems, which combine cells with synthetic systems to overcome biological hurdles. In brief, polymeric NPs can be cloaked with naturally derived cell membranes (Banskota et al., 2017). Fang et al. (2014) created a tumor vaccine (cancer cell membrane-coated nanoparticles) that is capable of presenting all the oncoantigens with correct orientation and enrichment while avoiding the housekeeping components of the cells. In their biohybrid NPs system, polymeric NPs were functionalized with cancer cell membrane vesicles lacking any intracellular components. By loading the NPs with a TLR-4 ligand and MPL, an adjuvant, they showed that it was possible to efficiently deliver tumor-specific antigens and stimulate a more robust anti-cancer immune response using such a biohybrid system when compared to using antigen-loaded NPs alone.

### Gold nanoparticles

GNPs are made in different sizes, shapes, and structures, depending on the application at hand. They are solid balls of gold and are made by the reduction of chloroauric acid, and their diameter varies from 5 to 100 nm. GNPs are useful for biomedical imaging and also for radiation dose enhancement (Zhang, 2015).

There are many cases that GNPs are used to improve the efficacy of tumor vaccine. Almeida et al. (2015) combined GNPs, OVA peptide antigen and CpG adjuvant together to verify enhanced therapeutic effect in a B16-OVA tumor model. He finally verified it and concluded that this enhanced therapeutic efficacy is likely due to the adjuvant effect of peptide-coated GNPs, as they induce inflammatory cytokine release when cultured with bone marrow DCs with the fact that free OVA and free CpG never induced immune response. In fact, the synergism of GNPs to tumor peptide vaccine has already been proved. Lin et al. (2013) in 2013 designed novel gold-based nanovaccines (AuNVs) using a simple self-assembling bottom-up conjugation method to generate high-peptide density delivery and more effective immune responses with limited toxicity. These high-peptide density AuNVs can stimulate CTLs better than free peptides and have great potential as carriers for various vaccine types.

In order to achieve better curative effect, the modification of GNPs and the combination of peptide has also been widely studied. For modification, in the study of Fytianos et al. (2015). GNPs were coated with PEG, polyvinyl alcohol (PVA) or a mixture of both with either positive or negative surface charge. The results suggested surface modification influenced uptake, due to limited uptake for PEG-COOH GNPs and high degree of internalization of (PEG + PVA)-NH2 and PVA-NH2 GNPS. Low uptake and high uptake also determine different biochemical responses. For combination, DeLong et al. (2009) combined DNA and RNA with protamine demonstrating association to GNPs. This complexes also exhibits very good antitumor activity.

In addition, GNPs have been successfully employed in inducing localized hyperthermia for the destruction of tumors or radiotherapy for cancer photodynamic therapy (Zhang, 2015). In this area, Hainfeld et al. (2004, 2010, 2013, 2014), have made a relatively comprehensive study and concluded GNPs absorb light, which lead to heat and ablate the tumor. Thus, GNPs can enhance the radiation therapy. And gold radiation enhancement significantly improved long-term survival compared with radiotherapy alone.

### Nanoemulsion

Nanoemulsion is a system of oil, water and amphiphile, which is a single optically isotropic and thermodynamically stable liquid solution. Thus, nanoemulsion is capable of encapsulating significant amounts of drugs. It can maintain stability and therapeutic effect of peptide. Compared to available traditional emulsions, nanoemulsion is characterized by its smaller size which is often 1–100 nm, higher efficiency, and elegant compatibility with tissues as well as with proteins.

Early in 2005, Ge’s et al. (2006) used nanoemulsion as delivery vehicle of the MAGE-1/heat shock protein 70/MAGE-3 (MHM) fusion protein vaccine and determined its humoral and cellular immune responses and the antitumor effects. MAGE represents the melanoma antigen. Heat shock proteins (HSPs) could conjugate proteins or peptides and improve their antigenicity. At the end, they summarized the MHM nanoemulsion can induce stronger cellular and humoral response and enhance more significant potency against the established MAGE-1-expressing or the MAGE-3-expressing tumors than the MHM fusion protein vaccine alone. Three years later, they still focused on this area. At this time, they encapsulated the MAGE1-HSP70 fusion protein and staphylococcal enterotoxins (SEA) complex protein in nanoemulsion as nanovaccine using magnetic ultrasound method. While this nanovaccine was via peroral administration route and could induce approximately similar antitumor immune responses to that via subcutaneous route and absolutely better than using protein alone (Ge et al., 2009).

However, there are few studies on nanoemulsion of tumor peptide vaccine in recent years. Because both the lack of stability and low efficiency of encapsulation limit the development of nanoemulsion.

### Other nano delivery systems

Here, we mentioned the nanogel and very small size proteoliposomes (VSSP).

For nanogel, Toyoda et al. (2015) examined the use of iontophoresis with cancer antigen gp-100 peptide-loaded nanogels for anti-cancer vaccination. Iontophoresis resulted in the accumulation of gp-100 peptide and nanogels in the epidermis. Moreover, tumor growth was suppressed by iontophoresis of the antigen peptide-loaded nanogels when compared to free peptides. Thus, iontophoresis of the antigen peptide-loaded nanogels serve as an effective transdermal delivery system for anti-cancer vaccination.

For VSSP, Caballero et al. (2017) raised a clinical research. Prostate castration-resistant carcinoma patients (*n* = 24) were intramuscularly vaccinated with human epidermal growth factor receptor-1 (HER1) vaccine consisting of the extracellular domain of HER1 molecule and VSSP from *Neisseria meningitidis*. Patients were included in five groups according to the vaccine dose. In result, no Grade III or IV adverse events were reported. High titers of anti-HER1 antibodies were observed in most of the evaluated patients. Only patients receiving the higher doses of vaccine showed significant tumor cell recognition and HER1 phosphorylation inhibition. The results suggested the HER1 vaccine is safe and effective. Morera et al. (2017) also raised a clinical research which focus on a cancer therapeutic vaccine. It based on recombinant-modified human vascular endothelial growth factor (VEGF) as antigen, encapsulated in VSSP. In result, the drug showed sufficient safety and it can prolong the survival time of patients.

## Conclusion and expectations

With the further research of molecular mechanism of tumorigenesis and the development of biotechnology, the means of tumor treatment are constantly improved and perfected. A growing number of experiments and clinical studies demonstrated that tumor immunotherapy is an effective anti-tumor treatment method (Le Boeuf et al., 2017; Zikry et al., 2017) and nano tumor peptide vaccines play an important role in anti-tumor immunotherapy (Kim et al., 2017). However, due to the weak immunogenicity of tumor autoantigen, the existence of tumor suppressive microenvironment and the immune escape induced by various factors, the development of tumor peptide vaccine faces great challenges. The wide application in preparation still has many unsolved problems.

**Figure 3.**
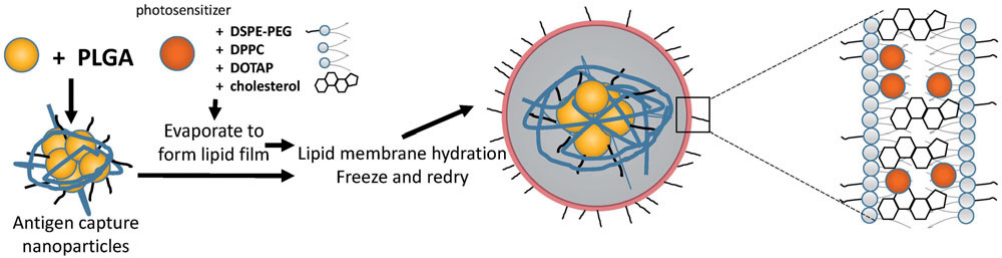
Idea of preparation of nanoparticle which make use of photosensitizer and also lead to immune response.

The application of nano drug delivery system to tumor vaccine has become the focus of anti-tumor vaccine research. It plays an important role in improving the immune effect of anti-tumor vaccine and overcoming the immunosuppression (Luo et al., 2015; Rahimian et al., 2015). While the research of nano drug delivery system to improve the anti-tumor effect of polypeptide vaccine is not yet proven, there are three possible ways to develop the nano tumor peptide vaccines, which are (i) looking for surfactant and cosurfactant with high efficiency and low toxicity, (ii) choosing the best method and the most suitable materials according to the characteristics of the drug to achieve high encapsulation efficiency and good release procedure *in vivo* and *in vitro*, like novel chitosan metal nanparticles which were illustrated a good tumor targeting, ability to load different hydrophobic anticancer drugs, and the ability to control the anticancer drug release rate (Ahmed & Aljaeid, 2016). And (iii) synthesizing and discovering new nontoxic, biocompatible, biodegradable polymeric pharmaceutical excipients. Finally, (iv) to open or find a new path or snap course by an innovative way, like nanoparticles coated with the membrane of a cell (Fang et al., 2014; Kroll et al., 2017). Specially, the blending of disciplines produces new research so that we should connect actively with other project like topical photodynamic therapy (PDT), which is also potential to treat cancer (Gomes et al., 2005). It is controllable by light with specific wavelength (Shi et al., 2017). And some nanostructured carriers in PDT is reported (Liu et al., 2016; Qidwai et al., 2016). Maybe we can add photosensitizer and tumor Ag and make the immune response more controllable than before (Figure 3).

We consider with the continuous optimization of nanomaterials, the development of nanotechnology, surface modification and functionalization of NPs, nano anti-tumor vaccine delivery system will be developed to provide technical platform and opportunities for the breakthrough of tumor vaccine, which has wide application value and great market prospect.
